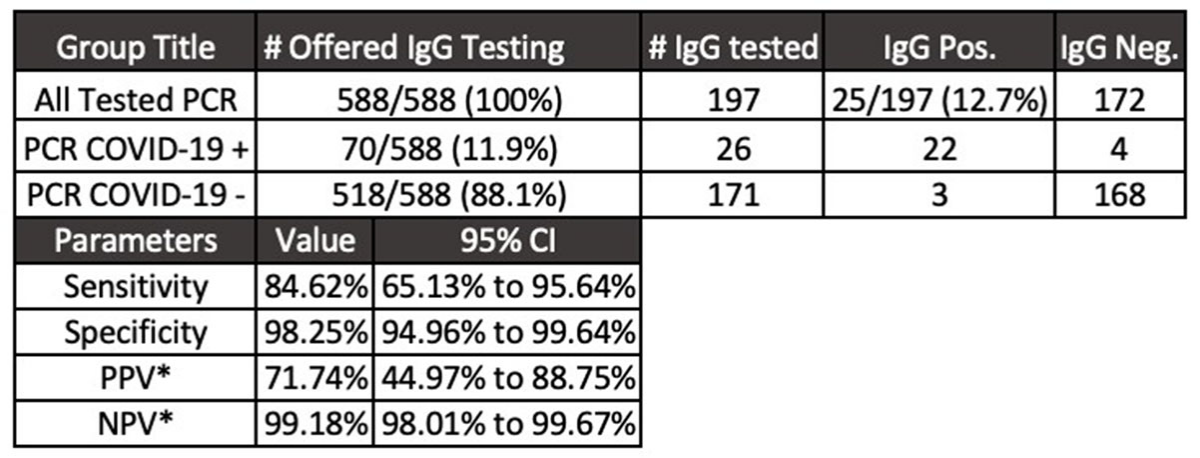# Use of COVID-19 Serologic Testing in Healthcare Workers with Acute Respiratory Tract Infection

**DOI:** 10.1017/ash.2021.81

**Published:** 2021-07-29

**Authors:** Amy Ray

## Abstract

**Background:** Diagnostic tests for COVID-19 are in high demand. Serologic assays are of interest as diagnostic adjuncts to SARS-CoV-2 quantitative polymerase chain reaction (PCR); however, many of the commercially available assays have limited validation data and clinical utility is unknown. We describe the utilization of SARS-CoV-2 IgG enzyme-linked immunosorbent assay (ELISA) for healthcare workers with acute respiratory tract infection (ARTI) who underwent SARS-CoV-2 PCR testing. **Methods:** The MetroHealth System is the largest public hospital system in Ohio, employing ~8,000 staff. COVID-19 detection began in early March 2020. EDI novel coronavirus COVID-19 IgG ELISA (KT-1032) targeting antibody response to viral nucleocapsid was obtained for diagnostic and seroprevalence analyses. Manufacturer reports of sensitivity and specificity of the assay are 100% and 99%, respectively. A 2-part test strategy for employees with symptoms of ARTI was implemented. Qualifying symptoms for SARS-CoV-2 PCR testing included fever and either cough or shortness of breath. Additional symptoms were included to reflect expanding knowledge of COVID-19. Employees who underwent SARS-CoV-2 PCR testing (Luminex ARIES) were offered serologic testing on day 14 following PCR result. Education accompanied the offer for serologic testing as well as the receipt of test result to aide interpretation. **Results:** From April 16, 2020, through July 6, 2020, 588 employees underwent PCR testing. Overall, 70 cases of COVID-19 were detected. Of the 197 employees who opted for serologic testing, IgG positivity was 12.6%. The mean time to IgG collection following PCR result was 30 days (range, 10–79). Using PCR results obtained in the clinical setting of ARTI as the diagnostic gold standard, IgG was 84.6% sensitive and 98.2% specific (Figure 1). **Conclusions:** In a population of symptomatic healthcare workers, SARS-CoV2 IgG testing was specific for COVID-19 diagnosis. Sensitivity was inadequate compared to the positive predictive agreement of 90% or greater required for US Food and Drug Administration emergency use authorization. In a low-prevalence environment for COVID-19 (<5%), a positive SARS-CoV-2 IgG has a low positive predictive value, which may falsely imply immunity and may negatively affect infection prevention practices.

**Funding:** No

**Disclosures:** None

**Figure 1.**